# Basal core promoters control the equilibrium between negative cofactor 2 and preinitiation complexes in human cells

**DOI:** 10.1186/gb-2010-11-3-r33

**Published:** 2010-03-15

**Authors:** Thomas K Albert, Korbinian Grote, Stefan Boeing, Michael Meisterernst

**Affiliations:** 1 Institute of Molecular Tumor Biology (IMTB), University of Muenster, Robert-Koch-Str. 43, 48149 Muenster, Germany; 2 Genomatix Software GmbH, Bayerstr. 85a, 80335 Munich, Germany

## Abstract

**Background:**

The general transcription factor TFIIB and its antagonist negative cofactor 2 (NC2) are hallmarks of RNA polymerase II (RNAPII) transcription. Both factors bind TATA box-binding protein (TBP) at promoters in a mutually exclusive manner. Dissociation of NC2 is thought to be followed by TFIIB association and subsequent preinitiation complex formation. TFIIB dissociates upon RNAPII promoter clearance, thereby providing a specific measure for steady-state preinitiation complex levels. As yet, genome-scale promoter mapping of human TFIIB has not been reported. It thus remains elusive how human core promoters contribute to preinitiation complex formation *in vivo*.

**Results:**

We compare target genes of TFIIB and NC2 in human B cells and analyze associated core promoter architectures. TFIIB occupancy is positively correlated with gene expression, with the vast majority of promoters being GC-rich and lacking defined core promoter elements. TATA elements, but not the previously *in vitro *defined TFIIB recognition elements, are enriched in some 4 to 5% of the genes. NC2 binds to a highly related target gene set. Nonetheless, subpopulations show strong variations in factor ratios: whereas high TFIIB/NC2 ratios select for promoters with focused start sites and conserved core elements, high NC2/TFIIB ratios correlate to multiple start-site promoters lacking defined core elements.

**Conclusions:**

TFIIB and NC2 are global players that occupy active genes. Preinitiation complex formation is independent of core elements at the majority of genes. TATA and TATA-like elements dictate TFIIB occupancy at a subset of genes. Biochemical data support a model in which preinitiation complex but not TBP-NC2 complex formation is regulated.

## Abstract 2

Genome-wide mapping of the human TFIIB and NC2 transcription factor binding sites reveals insights into the formation of the pre-initiation complex.

## Background

The core region of metazoan promoters shows various architectures and can harbor several distinct motifs, termed TATA box (TATA) [1], initiator (INR) [2], downstream promoter element (DPE) [3], downstream core element [4], upstream and downstream TFIIB recognition elements (BREu and BREd, respectively) [5,6] and motif ten element [7] (reviewed in [8]). These elements facilitate assembly of the transcription machinery in a cooperative manner and are thought to contribute to accurate initiation at a defined transcription start site (TSS) [9]. In a majority of vertebrate genes core promoter elements are less represented [10]. Instead, they reside in CpG islands and are GC-rich. These promoters assemble general transcription factors (GTFs) in a manner that remains poorly understood.

The general initiation factor TFIIB is absolutely required for transcription initiation by RNA polymerase II (RNAPII) [11]. TFIIB associates with TATA box-binding protein (TBP) and establishes sequence-specific contacts in the major groove upstream and in the minor groove downstream of TATA [12]. The upstream binding site, termed BREu, has been defined via an *in vitro *selection procedure employing the TATA-containing Adenovirus major late (AdML) promoter [6]. The corresponding high-affinity downstream element, BREd, was characterized via site selection in the context of the TATA-containing Adenovirus E4 (AdE4) promoter [5]. Both elements stabilize the TFIIB-TBP-promoter complex *in vitro*. BREu and BREd suppressed basal transcription of the AdML core promoter [13]; however, BREd enhanced activity of the AdE4 promoter [5]. Broadly, these data are in conflict with a general positive role of TFIIB in transcription.

The function of TFIIB has not been investigated *in vivo*, nor has TFIIB occupancy so far been correlated with gene activity. Prevalence of BREs in active genes remains subject to controversy. A computational study based on statistical analysis of curated promoter sets concluded that up to 25% of human core promoters contain a potential BREu. The motif was found to be enriched in CpG promoters (>30% frequency) but depleted in CpG-less promoters (<10% frequency) [14]. In contrast, a recent large-scale study of CAGE (cap analysis of gene expression) data sets in mammals did not reveal clear evidence of BREu over-representation in these regions [15]. The prevalence of BREd in mammalian promoters has not been investigated by bioinformatic means.

Genome-wide binding studies on general initiation factors have been extensively performed in yeast and include maps of TBP, TFIID and SAGA [16,17], GTFs [18], Mediator [19,20], and Mot1 and negative cofactor 2 (NC2) [21]. However, with few exceptions [22-25] comparable studies in mammalian cells are lacking. Here we conducted a comparative genome-wide analysis on promoter association of human TFIIB and NC2 and correlate it with gene expression and core promoter architecture. Whereas most genes direct preinitiation complexes (PICs) to their promoters in the apparent absence of core promoter elements, a small subset of highly expressed genes with high TFIIB/NC2 ratios direct binding of PICs via core promoters. Biochemical data suggest that TATA and regulatory factors positively control TFIIB but not (or to a lesser extent) NC2 binding, thereby providing a model for binding of GTFs in the absence of core elements and alterations in TFIIB/NC2 ratios inside cells. In addition to defining a library of promoters ranked by steady-state levels of PICs in human B cells, the comparative analyses of TFIIB and NC2 also establish a resource for human basal core promoters.

## Results

### Genome-wide promoter binding of TFIIB

**Figure 1:**
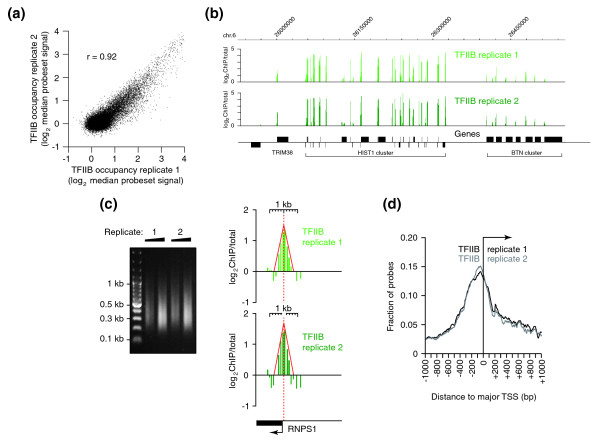
**Genome-wide promoter occupancy of TFIIB**. **(a)** TFIIB enrichment on human promoter arrays in two biological ChIP-chip replicates. Each spot represents the median of hybridization intensities obtained on 15 probes per individual promoter region (log2 scale). Pearson's correlation is denoted by r. **(b)** Signal tracks of the two TFIIB replicates for the *HIST1 *histone gene cluster and an adjacent *BTN *butyrophilin gene cluster on chromosome 6. Signals are bar-plotted as ChIP over non-enriched input DNA (ChIP/total) in log2 scale. **(c)** Resolution of ChIP-chip signals at a single gene promoter. The left panel shows the fragment length distribution of sheared ChIP DNA in the two replicates as determined by ethidium bromide staining of 250 ng (lanes 2 and 4) or 500 ng (lanes 3 and 5) of purified DNA loaded on a 1.4% agarose gel. Lane 1 is a DNA size marker with fragment lengths indicated on the left. The right panel shows magnified signal plots of the two TFIIB replicates at the *RNPS1 *promoter region. Scale is indicated at the top. The approximate width of the peak area is outlined in red, with the vertical hatched line denoting the peak center. The broken arrow marks the location and direction of the TSS. **(d)** Average binding profiles of the top 5% probesets for TFIIB replicate 1 (black line) and replicate 2 (grey line) relative to aligned TSSs at 10-bp resolution.

We conducted chromatin immunoprecipitation (ChIP)-chip analysis of TFIIB in two biological replicates from human B cell line LCL721 with promoter arrays covering roughly 24,000 TSS regions. Following binding site determination, an excellent overall correlation of the TFIIB ChIP-chip duplicates was observed (Pearson's correlation *r *= 0.92; Figure 1a). The concordance rate increased further for high-occupancy targets exhibiting the most intense hybridization signals; 97% (1,173 of 1,207) of promoters in the upper 5th percentile (95 to 100) of one replicate were found in the upper 10th percentile (90 to 100) from the other. On the level of individual promoter regions, TFIIB profiles also appeared largely identical. This is illustrated on extended gene loci such as the *HIST1 *histone gene cluster and the adjoining *BTN *butyrophilin gene cluster on chromosome 6 (Figure 1b), as well as on single promoter regions such as of the *RNPS1 *gene (Figure 1c). The latter also exemplifies the spatial resolution of single peak regions, which was approximately 300 to 400 bp and in good agreement with the median size of the bulk of sheared ChIP DNA. On a genome-wide scale, several thousands of binding sites were reproducibly detected when a peak finding algorithm [26] was applied to the two ChIP-chip samples (Table 1). To further substantiate resolution and reproducibility of the ChIP-chip data, average binding profiles of the two TFIIB samples were generated (Figure 1d). Probes from the upper 5th percentile of target promoters were remapped and plotted as relative fractions that are found in 10 bp intervals from aligned TSSs at +1. The replicates displayed a nearly identical Gaussian-type profile with peak maxima centered at position -50, thereby demonstrating high mapping accuracy in independent ChIP-chip samples. Moreover, the distance of TFIIB signals upstream of the TSS is in line with recent genome-wide ChIP data obtained for yeast TFIIB/Sua7 [18].

**Table 1:** TFIIB Peak Identification

	TFIIB replicate 1	TFIIB replicate 2
Peaks (mean + 1.0 s.d.)	4,139	4,713
Peaks (mean + 2.0 s.d.)	3,148	3,332
Peaks (mean + 2.5 s.d.)	2,371	2,493

Peaks in TFIIB ChIP-chip samples were identified by a triangular best-fitting algorithm (Mpeak) [26] using the indicated cut-offs for peak calls (s.d., standard deviation).

**Figure 2:**
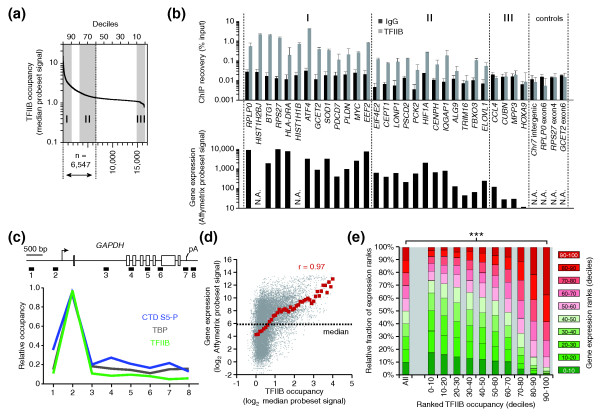
**Validation of TFIIB target promoters**. **(a) **Signal distribution of TFIIB enrichment. I, II and III denote groups of promoters from the upper 10th, 60th to 80th, or lower 10th percentile and correspond to high, mid-to-low, or no TFIIB occupancy. **(b) **Target gene validation. Selected genes from groups I to III were analyzed by ChIP-qPCR using TFIIB or IgG control antibody in a third chromatin sample from LCL721 cells in which two independent ChIP reactions were performed (upper panel). Genes are ordered from left to right according to TFIIB levels on the promoter arrays. The relative ChIP recovery is expressed as percentage of input (*y*-axis). The bars represent the mean, error bars the range of the two ChIP experiments. Corresponding gene expression levels in LCL721 B cells are shown in the lower panel. These were determined using Affymetrix U133 Plus 2.0 microarrays. They represent normalized hybridization signals of gene-specific microarray probesets. N.A., not analyzed. **(c) **Assembly of an active PIC at the *GAPDH *promoter. ChIP-qPCR was conducted with eight primer pairs spanning the human *GAPDH *locus (numbered boxes in top scheme). Results of ChIPs in LCL721 B cells with antibodies for TFIIB, TBP or the initiating form of RNAPII (CTD S5-P) are graphed as relative occupancy levels at the different amplicon locations (lower panel). **(d) **Scatter plot showing the genome-wide correlation of TFIIB binding to promoters (*x*-axis, log_2 _scale) and steady-state mRNA levels (*y*-axis, log_2 _scale) of the corresponding genes. The median of all expression array probesets with present calls is indicated by the dotted horizontal line. The red dots indicate the average expression in gene groups with increasing TFIIB occupancy. They were determined by moving a sliding window (step size 0.1) over the TFIIB data points and calculating the mean expression value for each increment. **(e) **Distribution of ranked gene expression quantiles (color-coding indicated to the right) in genes with increasing TFIIB occupancy levels. The difference in distributions was statistically evaluated using a Kolmogorov-Smirnov test (***P < 2e-16).

A subset of target genes was validated by quantitative ChIP-quantitative PCR (ChIP-qPCR) using a third independent B cell-derived chromatin sample. A total of 29 promoters were interrogated that represent high occupancy (group I, upper 10th percentile), mid-to-low occupancy (group II, 60th to 80th percentile), or no TFIIB occupancy (group III, lower 10th percentile) as determined by ChIP-chip (Figure 2a). Non-TSS regions were included as negative controls and a non-specific IgG ChIP served as background reference. With few exceptions, relative magnitudes of array signals were retained in the ChIP-qPCR analysis. Out of 25 promoters from groups I and II, 23 (92%) showed greater than 10-fold enrichment of TFIIB over control ChIP, proving them as true positives. Likewise, four of four group III promoters and four of four control regions were negative for TFIIB enrichment in ChIP-qPCR (Figure 2b). Based on the above confirmation rate, we estimate that approximately 6,000 (92% of 6,547) promoters - representing one-quarter of all 24,000 interrogated promoters - are bound by TFIIB. This is in line with previous estimates on the number of active promoters in human cells [25]. To corroborate specific promoter association of TFIIB, the glyceraldehyde 3-phosphate dehydrogenase gene (*GAPDH*) was scanned by ChIP-qPCR with eight primer pairs scattered throughout the locus (Figure 2c). Binding of TBP and the initiating form of RNAPII (phosphorylated at serine 5 in its carboxy-terminal domain) were monitored in parallel. All three factors showed pronounced binding to the *GAPDH *promoter, indicating assembly of an active PIC containing TFIIB. Similar results were obtained at other large gene loci (data not shown). TFIIB did not bind the 3' region of *GAPDH *or other genes [22] as was recently reported for yeast genes [27].

### TFIIB occupancy correlates positively with steady-state mRNA levels

At single genes TFIIB occupancy matched well with steady-state mRNA levels in LCL721 B cells [22] (Figure 2b, lower panel). To corroborate this at a genome-wide scale, TFIIB occupancy levels were correlated with mRNA levels for all genes. To this end, the median of the TFIIB ChIP-chip signal on each NimbleGen promoter array probeset was plotted against the normalized mRNA hybridization signal on the corresponding probeset of an Affymetrix gene expression array (Figure 2d). Then, a sliding window was moved over the ChIP-chip data from genes with low TFIIB levels to genes with high TFIIB levels and the average expression for these subgroups was determined. The resulting curve revealed a significant positive correlation between TFIIB occupancy and gene expression (Pearson's correlation *r *= 0.97). Moreover, a disproportionately high number of the most strongly expressed genes bear high TFIIB levels, as revealed by the skewed distribution of expression quantiles (Figure 2e). Here, 94% of the genes in the upper 10th percentile of TFIIB occupancy are expressed above average (median of all expression array signals), and 37% fall into the top 10% of expressed genes. In contrast, 26% of the genes in the lower 10th percentile of TFIIB occupancy are expressed above average, and only 2% of those are amongst the top 10% of all expressed genes. These outliers may reflect gene expression control at posttranscriptional stages, for example, through stabilization of mRNAs. To statistically evaluate the observed difference, a Kolmogorov-Smirnov test was applied. It confirmed with a significance level of *P *< 2e-16 that the distribution of expression signals in the upper 10th percentile of TFIIB occupancy is highly dissimilar to the distribution in all genes. Taken together, these analyses indicate that TFIIB-dependent PIC formation provides an excellent measure for gene activity, both at the single gene and the genome-wide level.

### Human core promoter structure associated with preinitiation complexes

**Figure 3:**
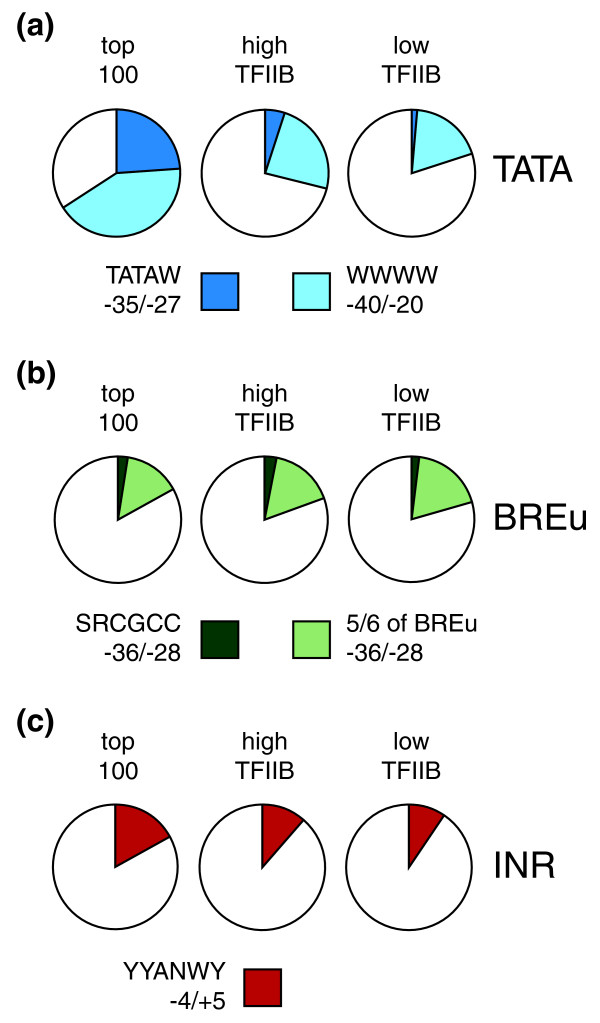
**Frequencies of core promoter elements in TFIIB target promoters**. Pie charts showing the relative frequencies of **(a) **TATA and TATA-like motifs, **(b) **BREu, and **(c) **INR consensus in the top 100 TFIIB-bound promoters (left chart), high-TFIIB promoters (middle chart) and low-TFIIB promoters (right chart). Motif sequences and positions that were requested for a hit are shown below the charts.

General features of high-TFIIB promoters (upper 5th percentile) were compared to low-TFIIB promoters (45th to 50th percentile) and no-TFIIB promoters (lower 5th percentile). For each group the core promoter sequences from position -50 to +50 were extracted, aligned at the major TSS and represented in a nucleotide frequency plot [28] (Additional file 1). High- and low-TFIIB promoters have 61% and 62% GC content compared to 54% of the no-TFIIB promoter set, well above the 38% for the whole human genome [10]. An exception is the region surrounding the TSS, where, consistent with previous CAGE data, pyrimidine (Py) at -1 and purine (Pu) at +1 (with G as the most frequently base at +1) is seen [15]. We next searched for core promoter elements in the different promoter groups, including a block of 100 genes with the highest levels of TFIIB binding. In the latter group, referred to as 'top 100', 24% of all promoters contained a TATA consensus motif (TATAW, with the first T at position -31 relative to the TSS). The number decreased in the less frequently bound groups, reaching an overall 5% in high-TFIIB and 1.4% in low-TFIIB promoters (Figure 3a). TATA-like sequences (WWWW) within position -20 to -40 were found in 66% of the top 100 genes, and decreased to 29% and 20% in high- and low-TFIIB promoters (Figure 3a). In contrast, the frequency of the BREu motif (SRCGCC positioned immediately upstream of TATA) was around 2 to 3% and independent of TFIIB occupancy (Figure 3b). Relaxation of the BREu sequence constraints by allowing for one mismatch elevated frequencies to 17%, 19% and 21% in the top 100, high-TFIIB and low-TFIIB genes, respectively. Thus, unlike TATA, BREu and BREu-like sequences do not correlate with TFIIB occupancy. For BREd, we analyzed only TATA consensus promoters within high-TFIIB promoters to allow accurate location of the motif downstream of TATA as described [5]. Despite its degenerated consensus (RTDKKKK) we did not find a single TATA promoter containing a full match to this sequence in this subgroup. Allowing for one mismatch did not reveal enrichment of BREd above stochastic levels. Hence, BREd is essentially absent in TATA consensus promoters with high TFIIB levels. Finally, we found that 17% of the top 100 genes contained a full match to the initiator sequence (YYANWY) around the TSS (with the central A between position -4 and +5). INR frequency was slightly decreased in high-TFIIB promoters (12%) and low-TFIIB genes (10%) (Figure 3c). Like TATA, initiator was readily discovered using the *ab initio *motif discovery program MEME [29] in the top 100 TFIIB-bound promoters (data not shown). No other motifs with reasonable *E*-values (measuring significance of enrichment) and/or specific positioning in the core region could be identified. Most notably, MEME uncover neither BREu nor BREd from these TFIIB-bound promoters.

### Comparison of genome-wide TFIIB and NC2 promoter occupancy

**Figure 4:**
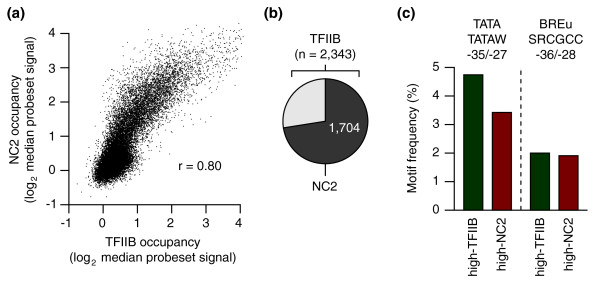
**TFIIB versus NC2 binding to human promoters**. **(a) **Genome-wide correlation of TFIIB and NC2 binding levels on promoter regions. *r*, Pearson's correlation. **(b) **Pie chart showing the overlap of high-occupancy promoters (upper 10th percentile) recovered in TFIIB and NC2 ChIP-chip samples. **(c) **Comparison of the frequencies of TATA and BREu consensus sequences in high-TFIIB versus high-NC2 promoters.

NC2 ChIP-chip was conducted in parallel to TFIIB and as described previously [22]. The two data sets proved to be closely related (Pearson's coefficient of 0.8; Figure 4a). Nearly three-quarters of TFIIB target promoters from the upper 10th percentile were also identified in the upper 10th percentile of NC2 targets (Figure 4b). Binding of the repressor NC2 to active genes and overlap in targets is not unexpected given that both factors target exclusively active genes bound by TBP. TATA frequency was slightly higher in TFIIB target promoters (4.8% versus 3.3% in NC2 target promoters), whereas BREu frequency in the two sets was identical (Figure 4c). The limited preference of NC2 for TATA confirms previous biochemical analyses conducted on model promoters *in vitro *([30]; see also below).

### Intact core promoters select for TFIIB and against NC2

**Figure 5:**
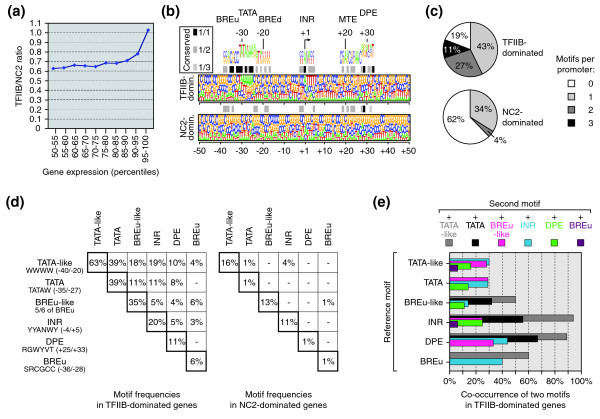
**High TFIIB/NC2 ratios select for TATA and combinations of TATA with other core promoter elements**. **(a) **Correlation of TFIIB/NC2 ratio to gene expression. Genes were grouped into percentiles of expression levels (*x*-axis). For each group, the mean value of TFIIB or NC2 occupancy on all promoters within this group was determined. From these values the ratio was calculated and is plotted as a blue curve in the graph. **(b) **Nucleotide frequency plots [28] of the top 100 TFIIB-dominated genes (upper panel) or the top 100 NC2-dominated genes (lower panel). Core promoter sequences from position -50 to +50 were extracted and aligned at the TSS (broken arrow). Letter heights reflect relative base frequencies at the given position. Shaded boxes on top of each panel indicate matches to the consensus sequences of core promoter elements shown above. **(c) **Pie charts depicting the percentage of promoters of either TFIIB-dominated genes or NC2-dominated genes that contain zero, one, two, or three motifs in their core region. **(d) **Matrix showing absolute frequencies of the indicated core promoter motifs or motif combinations in TFIIB-dominated genes (left) or NC2-dominated genes (right). **(e) **Synergistic motif combinations in core promoters of TFIIB-dominated genes. The bar graph depicts how often one of the specified reference motifs is found in combination with a second motif in the same promoter. Co-occurrence of two motifs is expressed as fractional percentage, with the reference motif alone set to 100%.

Differences in the underlying gene architectures may favor PIC formation (that is, TFIIB-TBP) versus PIC inhibition (that is, NC2-TBP) *in vivo*. To address this, we sought to relate the relative occupancy levels of TFIIB versus NC2 on core promoters with the mRNA output of the corresponding genes. To this end, relative factor occupancy levels were calculated for percentile-ranked gene expression groups by determining the mean enrichment of either TFIIB or NC2 on all genes within a given expression quantile. The ratio of these two values was built and is plotted in Figure 5a. It is informative for steady-state PIC levels on promoters. The majority of genes displayed a uniform TFIIB/NC2 ratio at their promoters, reasoning against tight control of binding of either one. However, the TFIIB/NC2 ratio increased steeply towards the most highly expressed genes (that is, in the 90th to 95th and 95th to 100th percentiles). The overall range of TFIIB/NC2 ratios on individual gene promoters was between 3.0 (where TFIIB and PIC is dominating) and 0.12 (where NC2 is dominating).

We then asked if we could identify core promoter structures that relate to different TFIIB/NC2 ratios. Here, we focused on active genes, that is, genes that are expressed above average and are bound by both factors, using the 60th percentile for TFIIB and NC2 gene occupancy as well as for steady-state mRNA levels as cut-off. From these, the top 100 genes showing the highest or lowest TFIIB/NC2 ratios were selected for further analysis. Alignment of the promoter regions of the top 100 TFIIB-dominated genes yielded structured core regions with the most frequent bases resembling the INR consensus at positions -2 to +5 (Figure 5b, upper panel). Preferred bases at positions -35 to -25 (CGGCTAAAAAA) matched conserved BREu and TATA residues. Also, a G-rich sequence around +30 (GGGCGT) resembled the DPE motif (RGWYVT) [3] identified in *Drosophila*. In contrast, alignment of NC2-dominated genes did not reveal recognizable core elements. Instead, the core regions of these genes were enriched for G and C, which were the most frequent bases at every single position from -50 to +50 (Figure 5b, lower panel).

The enrichment of core promoter elements in TFIIB- versus NC2-dominated genes was analyzed further. Enumeration of motif frequencies revealed that in 81% of TFIIB-dominated genes but in only 38% of NC2-dominated genes, at least one core promoter motif was present (Figure 5c). Strikingly, 27% and 11% genes of the former group harbored combinations of two or three motifs, whereas only 4% and zero genes of the latter group contained such binary and ternary motif combinations. Individual motif frequencies are summarized in Figure 5d. Comparing TFIIB- versus NC2-dominated genes, TATA was revealed as the most strongly enriched motif. It was present in 39% of TFIIB-dominated genes but in only 1% of NC2-dominated genes (Figure 5d). Other significantly enriched motifs included DPE (11% versus 1%), BREu (6% versus 1%) and, to a lesser extent, TATA-like (63% versus 16%), BREu-like (35% versus 13%) and INR (20% versus 13%). Again, BREd was not identified above stochastic levels in the TATA downstream region. In aligned TATA consensus promoters of TFIIB-dominated genes, the preferred bases upstream of TATA were consistent with described TFIIB contacts [6,12] at the BREu (G at position -34 and C at position -32 were found in 42% and 52% of all TATA promoters), whereas the base composition downstream of TATA did not show homology to the BREd consensus. For example, thymine was the least frequent base at position -24, while it is the most frequent base in the *in vitro *selected BREd consensus sequence RTDKKKK [5]. Base composition rather resembled the upstream region by showing preferential usage of G and C. From these data and the insignificant abundance of BREd, we conclude that BREd does not correlate with PIC formation and TFIIB binding *in vivo*.

The high prevalence for the occurrence of motif combinations in TFIIB-dominated genes in illustrated in Figure 5e. In line with the known synergy between INR and TATA [14], 94% and 56% of promoters harboring the INR motif also contained a TATA-like or TATA consensus sequence, respectively. A strong linkage was also observed for DPE and TATA: 89% of DPE promoters harbored a TATA-like sequence, and 67% of DPE promoters a TATA consensus motif in the upstream region around -30. This is unexpected, since the DPE was functionally identified in *Drosophila *promoters as a surrogate core element in TATA-less promoters [3]. Finally, 50% of promoters with a BREu-like motif around position -32 contained an adjacent TATA-like sequence and 32% a downstream TATA consensus, reflecting the above observation of conserved BREu residues in TATA-containing promoters with high-TFIIB levels. Taken together, TFIIB strongly selects for TATA as well as for synergistic combinations of TATA with INR or DPE and, to a lesser extent, with BREu-like sequences in human core promoters.

### NC2 is more frequent on genes with multiple start sites lacking defined core promoter elements

**Figure 6:**
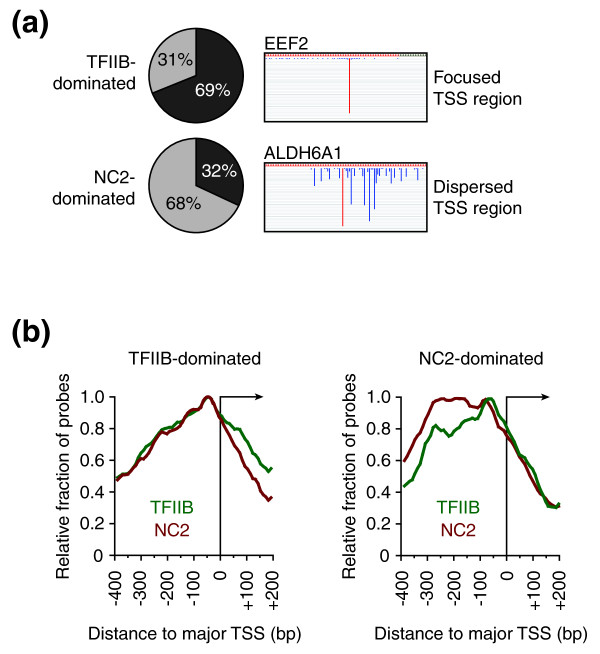
**TFIIB/NC2 ratio reflects transcription start site patterns**. **(a) **Start site patterns in TFIIB- versus NC2-dominated genes. Pie charts show the fraction of promoters for which a distinct TSS pattern could be assigned. Individual regions displaying single peak or dominant peak shape were classified as focused TSSs, and those displaying broad or multimodal peak shape were classified as dispersed TSSs (classification following [43]). Examples of genes with focused and dispersed TSS patterns (taken from [44]) are shown. **(b) **TFIIB profiles (green) and NC2 profiles (red) at promoters of TFIIB-dominated genes (left), or NC2-dominated genes (right). For each profile the relative fraction of high-score (upper 5th percentile) probes mapping to distinct 10 bp bins around the aligned TSS is plotted, with score maxima arbitrarily set to 1.

Next, CAGE data [31] were compared with the top 100 of either TFIIB- or NC2-dominated genes. This analysis revealed that the majority of TFIIB-dominated genes (69%) displayed focused TSS patterns starting from one or very few dominant sites (Figure 6a). At NC2-dominated promoters, dispersed TSS distributions were enriched (68%). CAGE tags provide a quantitative measure for mRNA abundance. TFIIB-dominated genes contained, on average, 948 CAGE tags per cluster, whereas this number decreased to 279 tags per cluster for NC2-dominated genes.

Average occupancy profiles of TFIIB and NC2 at promoters of genes with high or low TFIIB/NC2 ratios (1,000 for each group) showed similar factor profiles at the former group, with peak maxima coinciding at position -50 (Figure 6b, left). In contrast, at genes with low NC2/TFIIB ratios a broader distribution of both factors (ranging from -90 to -290) was observed (Figure 6b, right). Here, NC2 is markedly enriched in upstream regions relative to TFIIB, perhaps indicating a specific role of NC2 on genes with multiple start sites. The relevance of the difference in TFIIB versus NC2 distributions on these genes was confirmed with high confidence (*P *< 2.2e-16) by running a Wilcoxon-Mann-Whitney test on the positions of TFIIB and NC2.

### TFIIB/NC2 ratios are influenced by both activators and core promoter elements

**Figure 7:**
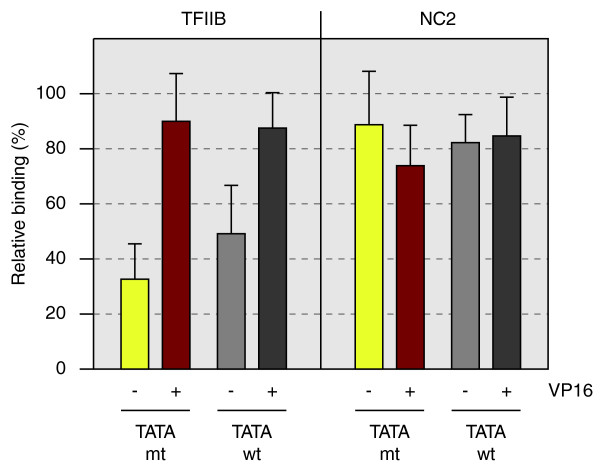
**TFIIB and NC2 binding to TATA (+/-) promoters in nuclear extracts**. PICs were formed on immobilized HIV/AdML promoter templates containing a wild-type (wt) or mutant (mt) TATA box using Jurkat nuclear extract under basal conditions (-VP16) or in the presence of the activator Gal4-VP16 (+VP16). After washing, the reactions were analyzed by immunoblotting with specific antibodies against TFIIB or NC2. Blots were scanned and quantified using ImageJ [41]. Bars and error bars represent mean and standard deviation of three independent reactions. TFIIB and NC2 template association is expressed as percentage of relative binding, with the reaction showing maximum binding set to 100%.

To this point our data suggested that core promoters and specifically TATA in synergy with other elements influence the equilibrium between TFIIB and NC2. On the other hand, PICs form in the absence of core elements in the majority of genes, raising questions as to how factors are directed here. To model this situation, we employed an *in vitro *PIC formation assay in which transcription complexes were assembled on a Gal4-responsive heterologous promoter template containing either a wild-type or mutant TATA box, both in the presence and absence of the model activator Gal4-VP16 (Figure 7). Whereas the activator enhanced TFIIB binding, NC2 remained essentially irresponsive to at least this activator. Notably, the positive activator effect on TFIIB was stronger for the template containing a mutant TATA element (three-fold increase of TFIIB binding) compared to the wild-type TATA template (1.8-fold increase of TFIIB binding).

## Discussion

Our analysis establishes the first genome-wide reference data set for steady-state occupancy levels of vertebrate PICs. The comparative analysis of TFIIB and NC2 occupancy with gene expression further provides a framework for future detailed analyses of basal versus gene regulatory mechanisms on individual or groups of human genes. Our data presently suggest that PIC (or TBP-TFIIB) association correlates with TATA or is independent of core elements altogether, whereas NC2 association is largely independent of the underlying core promoter structure.

TFIIB and NC2 act globally and are present at active genes. We report a strong positive correlation of TFIIB with gene expression levels at a genome-wide scale (Figure 2d), which is in line with the factor's original definition as a crucial PIC component [11]. Conflicting reports indicating a negative TFIIB impact through BRE interactions (see Introduction) are not represented in our genome-wide data, although we can not exclude such mechanisms at specific genes. At least for highly expressed genes, our data reason for an inhibitory function of NC2. It remains to be proven that NC2 can also act positively on certain genes. Candidates for the latter are multiple start sites genes that produce high mRNA levels and display high NC2/TFIIB ratios. A possible mechanism is that efficient promoter association of TBP depends on NC2 at such genes.

Our data reason against a positive influence of core elements on NC2 promoter association. For example, NC2-dominated genes with high NC2/TFIIB ratios were enriched for GC but depleted for core promoter elements, in particular TATA, BREu and DPE (Figure 5b-d). Attempts to show direct specificity of TBP-NC2 complexes for GC-rich regions failed (Christine Göbel and MM, unpublished). Enrichment of NC2 on such genes probably reflects low initiation rates from start sites located further upstream of a major TSS. At the majority of genes, however, TFIIB and NC2 occupancy distribution is very similar. This indirectly suggests that TBP, the partner of both TFIIB and NC2, dictates the recognition site. However, alternative scenarios in which NC2 binding and PIC formation become coupled could be projected. For example, when RNAPII clears the promoter it leaves TBP behind [32]. The latter may subsequently be recognized and stabilized by the abundant NC2 complex.

NC2 occupancy and activity appear in a distinct light if compared with TFIIB. A generally positive correlation of binding with the presence of TATA turns into a negative correlation relative to the competing GTF TFIIB. Related to this, NC2 occupancy positively correlates with gene expression, yet TFIIB correlation with it is more pronounced. Indeed, TFIIB/NC2 ratios increase especially in the most strongly expressed 5% of the B cell genes (Figure 5a). Our data thus reason for a negative role of NC2 at strongly expressed genes carrying intact core promoters. This is consistent with the original reports by Reinberg and our laboratory [33-36].

TATA, although a rather infrequent motif, is positively correlated with the binding of TFIIB (Figure 3a). Somewhat surprisingly, we found little evidence for a critical role of the previously defined BREs in PIC formation. The BREu consensus is found in approximately 3% of the preferred TFIIB target genes (Figure 3b). In pre-selected TFIIB-dominated genes the BREu frequency increases only moderately to 6% (Figure 5c). BREd is not found above stochastic levels and, hence, is apparently not linked to TFIIB-driven PIC formation. One may object that BREs are more degenerated in sequence and difficult to track, especially in the absence of TATA boxes, where the position of TFIIB-DNA interaction is less predictable. Along this line we note that genes with a high TFIIB/NC2 ratio often carry GC-rich regions that resemble the upstream BREu. In summary, the data imply that conserved BRE motifs with position and sequence fidelity comparable to the TATA consensus do not play a significant role in TFIIB promoter association.

Most genes that bind TFIIB with high efficiency (top 5%) seem not to employ core elements to facilitate or stabilize GTF-core promoter interactions. TATA consensus is found with a frequency below 5%, TATA-like elements reach 29% (Figure 3a). The DPE, downstream core element and motif ten element were not detected above stochastic levels in the top 5% of target genes of either TFIIB or NC2. So far our attempts have failed to select associated structure in core promoters for the few genes where these elements may play a role. We could also not reconstruct an alternative (that is, mammalian) DPE from the information obtained with high-TFIIB or high-NC2 target genes. Generally, core elements were most well represented in a small subset of genes that have high expression levels and at the same time display high TFIIB/NC2 ratios. In this small subset we did identify with a frequency of 11% a positioned DPE-like motif conforming to the *Drosophila *consensus RGWYVT [3]. In contrast to the situation in *Drosophila*, DPE presence is strongly linked to TATA in this subset of human promoters (Figure 5d).

We hypothesize that at the majority of genes lacking intact core elements, promoters are accessible in chromatin and/or may ultimately direct GTFs to promoters via interactions with regulatory surfaces, for example, through gene-specific activators. To prove this assumption, individual genes will have to be studied in detail both *in vivo *and *in vitro*. While this will undoubtedly uncover different scenarios in directing PIC formation, we have initially taken a reductionist biochemical approach using one model activator together with prototypic (TATA+/-, INR+) promoters (Figure 7). Most importantly, the activator, and to a lesser extent TATA, influence binding of TFIIB, while NC2 is unresponsive to the activator. NC2 also has less affinity for TATA, yet TBP-NC2 complexes retain moderate specificity for TATA [30]. This result suggests that PICs might be directed to promoters by activators, whereas the core promoters contribute to their binding and less to the association of NC2 with promoters. The high prevalence of intact core elements and their combinations in the small subset of TFIIB-dominated genes as well as the positive correlation of high TFIIB/NC2 ratios to gene expression levels (Figure 5a) suggests that core promoter elements contribute to gene activity in this subgroup of genes. The model predicts that binding of GTFs may be largely directed by activators on GC-rich promoters, whereas direct binding of GTFs and, to a lesser extent, regulatory factors contribute to the activity of the small subset of genes carrying multiple intact core elements within promoters.

## Conclusions

TFIIB and NC2 are global factors acting at a large fraction of all human genes. TATA was revealed as the most influential element for TFIIB recruitment and PIC formation. Most genes, however, recruit general factors in the absence of known GTF binding sites. We hypothesize that at these genes, TFIIB/NC2 ratios are determined by interactions between regulatory factors and the RNAPII machinery. There is overwhelming evidence for the influence of regulatory factors on PIC formation, but little precedence for direct action of activators on NC2. This is also the result of our *in vitro *binding studies using VP16 as a model for transactivators. On the other hand, core promoter elements are the major determinant for PIC binding in a subgroup of highly expressed genes that are characterized by high TFIIB/NC2 ratios. This subgroup establishes a small pool of human core promoters that may prove useful for future analyses of interactions between GTFs, cofactors and core promoters.

## Materials and methods

### Antibodies

Anti-TFIIB antibody (sc-225) and non-specific IgG serum (sc-2027) were purchased from Santa Cruz Biotechnology (Santa Cruz, CA, USA). Anti-NC2 alpha (DRAP1) antibody 4G7 has been previously described [22].

### Cell culture

LCL721 cells were grown in RPMI 1640 medium supplemented with 10% (v/v) heat-inactivated fetal bovine serum, 5 mM L-glutamine and 100 units/ml penicillin-streptomycin (all from Invitrogen, Karlsruhe, Germany) in a humidified incubator at 37°C and 5% CO_2_.

### Chromatin immunoprecipitation

We pelleted 1 × 10^8 ^cells (0.4 × 10^6 ^cells/ml) by centrifugation (1,200 rpm, 5 minutes) and washed them with PBS. The cell pellet was resuspended in 36 ml of PBS. Cells were fixed by adding 4 ml of a freshly prepared 10% formaldehyde solution (10% (v/v) formaldehyde (Sigma-Aldrich, Taufkirchen, Germany), 140 mM NaCl, 1 mM EDTA, 0.5 mM EGTA, 50 mM Hepes-KOH pH 8.0). Cross-linking was done for 9 minutes at room temperature, followed by quenching with 125 mM glycine, with immediate transfer of cells to ice followed by 5 minutes incubation on ice. Cells were washed twice with ice-cold PBS and sequentially lysed by resuspending the cell pellet in 5 ml of ice-cold ChIP lysis buffer 1 (50 mM Hepes-KOH pH 7.4, 140 mM NaCl, 1 mM EDTA, 0.5 mM EGTA, 10% (v/v) glycerol, 0.5% (v/v) Igepal CA-630 (Sigma-Aldrich, Taufkirchen, Germany), 0.25% Triton X-100 (Sigma-Aldrich), and freshly added 1× protease inhibitor cocktail (Roche, Mannheim, Germany)) and 10 minutes rotation at 4°C. Cells were collected by centrifugation (4,000 rpm, 10 minutes, 4°C), followed by resuspension in 5 ml of ice-cold ChIP lysis buffer 2 (10 mM Tris-HCl pH 8.0, 200 mM NaCl, 1 mM EDTA, 0.5 mM EGTA, freshly added 1× protease inhibitor cocktail) and 10 minutes rotation at 4°C. After centrifugation (4,000 rpm, 10 minutes, 4°C), the pellet was resuspended in 3 ml of ice-cold ChIP lysis buffer 3 (10 mM Tris-HCl pH 8.0, 140 mM NaCl, 1 mM EDTA, 1 mM EGTA, 0.5% N-lauryl sarcosine (Sigma-Aldrich), 0.1% sodium deoxycholate (Sigma-Aldrich), and 1× protease inhibitor cocktail). Acid-washed glass beads (212 to 300 microns; Sigma-Aldrich) were added, and the cross-linked chromatin was sheared to an average size of 300 bp by 6 minutes sonication (40% power output, with pulses set to 30 s ON/10 s OFF) in an ice-water bath using a Branson 250-D sonicator and a microtip. After sonication, Triton X-100 was added to 0.5% as final concentration, and the lysate was centrifuged (5,500 rpm, 5 minutes, 4°C) to remove cell debris. The chromatin extract was pre-cleared with 100 μl blocked (pre-absorbed with PBS/0.5% (w/v) BSA (Sigma-Aldrich)) protein A/G sepharose FF beads (GE Healthcare, Munich, Germany) for 2 h at 4°C, quantified in a UV spectrophotometer and diluted to 1 mg/ml and 0.25% N-lauryl sarcosine. We used 500 μl of the chromatin extract per single ChIP reaction in lubricated tubes in a total volume of 1 ml. The extract was incubated overnight at 4°C with 50 μl blocked protein A/G sepharose beads that had been pre-adsorbed with 10 μg of antibody. Immune complexes were collected by centrifugation (3,000 rpm, 1 minute, 4°C) and washed six times with 1 ml of ice-cold ChIP wash buffer (50 mM Hepes-KOH pH 7.4, 500 mM LiCl, 1 mM EDTA, 1% Igepal Ca-630, 0.7% sodium deoxycholate, and freshly added 0.5× protease inhibitor cocktail) and one time with 1× TE (10 mM Tris-HCl pH 8.0, 1 mM EDTA) containing 50 mM NaCl. The protein-DNA complexes were eluted from the beads by adding 200 μl ChIP elution buffer (50 mM Tris-HCl pH 8.0, 10 mM EDTA, 1% sodium dodecyl sulfate (SDS; Invitrogen)) and incubation at 65°C under constant agitation for 10 minutes. After removal of beads by centrifugation (6,000 rpm, 5 minutes, room temperature) the supernatant was incubated at 65°C overnight to revert the cross-links. Then, the sample was diluted to 400 μl with 1× TE. DNAse-free RNAse A (8 μg; RPA grade; Applied Biosystems, Foster City, CA, USA) was added and the sample was incubated for 1 h at 37°C, followed by addition of proteinase K (PCR grade; Roche) to 250 μg/ml and digestion for 2 h at 55°C. Genomic DNA was isolated from the precipitated material as well as from the sheared chromatin input (1% of the material used for ChIP) by phenol extraction and ethanol precipitation.

### ChIP-chip

ChIP and input DNA was end-polished using T4 DNA polymerase (New England Biolabs, Ipswich, MA, USA) and 200 μM dNTPs for 20 minutes at 12°C. After phenol extraction and ethanol precipitation, blunted DNA was ligated to 100 pmol of annealed linker (of oligo-25, 5'-GCGGTGACCCGGGAGATCTGAATTC, and oligo-11, 5'-GAATTCAGATC) using T4 DNA ligase (New England Biolabs) overnight at 16°C. DNA was ethanol precipitated and amplified by ligation-mediated PCR in a total volume of 55 μl containing 250 μM dNTPs, 50 pmol of oligo-25, 5 units of Taq polymerase (New England Biolabs) and 0.025 units of Pfu Turbo polymerase (Stratagene, La Jolla, CA, USA) for one initial cycle consisting of 2 minutes at 55° (during which polymerase was added), 5 minutes at 72°C and 2 minutes at 94°C, followed by 22 cycles of 0.5 minutes at 94°C, 0.5 minutes at 60°C and 2 minutes at 72°C, and a final 4-minute extension at 72°C. Amplicons were purified with a PCR purification kit (Qiagen, Hilden, Germany). At least 5 μg of amplified ChIP and input DNA were labeled and hybridized to human promoter arrays (NimbleGen HGS17 human 1.5 K promoter chip) containing 24,134 human promoter regions, each represented by a probe set of 15 tiling 50-mer oligonucleotide probes covering 1.5 kb DNA around TSSs. Slides were scanned by NimbleScan and raw data were processed by NimbleGen according to standard procedures [37]. Briefly, for each feature a log2 ratio of the hybridization intensities of the co-hybridized ChIP and input DNA was determined. These ratios were scaled to center the data around zero by robust statistics. Specifically, scaling was performed by subtracting Tukey's bi-weight mean for the log2 ratios of all array features from each individual log2 ratio. The median of the scaled log2 ChIP/input ratio of each probe set provides a measure for promoter occupancy.

### ChIP-qPCR

ChIP-qPCR analysis was done with Power SYBR green PCR master mix in an ABI StepOne Plus thermocycler (Applied Biosystems, Foster City, CA, USA). Triplicate reactions were carried out in a total volume of 10 μl containing 4 pmol of forward and reverse primers. Reactions containing serially diluted input DNA were used as standard curve to quantify ChIP DNA reactions. Melting curve analysis was used to determine the specificity of all reactions. Primer sequences are available upon request.

### Computational and statistical analyses

Peak finding was done using the Mpeak program [26]. Peaks were called under different stringency settings, with cut-offs of mean log_2 _signal ratios (ChIP versus total DNA) plus either 1, 2, or 2.5 standard deviations (Table 1). High-resolution binding profiles were generated by extracting and remapping of relevant array probe sequences to their exact position in the NCBI build 36 of the human genome. A slightly modified version of the RegionMiner software (Genomatix, Munich, Germany) was used to correlate the position of single high-score probes (upper 5th percentile) with annotated TSSs (Figures 1d and 6b). To avoid positional bias, the relative fraction of high-score probes mapping to distinct 10-bp bins around aligned TSSs was calculated with respect to the number of all available probes at this position. Correlation analyses of factor occupancy and gene expression levels were conducted as follows: steady-state mRNA expression levels were derived from polyadenylated RNA of LCL721 cells hybridized to an Affymetrix U133 Plus 2.0 microarray that covers probesets for the analysis of over 47,000 human transcripts (data available as SI Data Set 2 in reference [22]). Data analysis with GCOS software and default statistical algorithm parameters was performed by Affymetrix service provider KFB (Regensburg, Germany). Log_2_-scaled ChIP/input enrichment of TFIIB or NC2 on NimbleGen promoter probesets (see above) were matched with corresponding Affymetrix probeset IDs (only using probesets with 'present' calls) to generate the gene expression correlation analysis of TFIIB (Figure 2d) or TFIIB/NC2 ratios (Figure 5a). Degree of correlation between data sets in Figures 1a, 2d and 4a was determined by applying the Pearson correlation function in Microsoft Excel. For other statistical analyses the Bioconductor package was used. These included the evaluation of the statistical significance of the difference of Affymetrix probeset signal values for genes that do have a top 10% enrichment score against the distribution of all probeset signal values using a Kolmogorov-Smirnov test (Figure 2e). The hypothesis that both distributions are similar can be denied on a significance level of *P *< 0.01, thus making it clear that the data for these genes differ from the complete set. To accomplish this we used the ks.boot function from the 'Matching' package (provided by JS Sekhon) [38], for the R software for statistical computing [39]. Similarly, the relevance of the difference in TFIIB versus NC2 distributions on genes (Figure 6b) was revealed by running a Mann-Whitney-Wilcoxon test on the factor positions using the R package [39].

### Immobilized template assay and *in vitro *transcription

Immobilized template assays and *in vitro *transcription were performed as described [40]. The pGL2-MRG5 promoter template was amplified from vector pGL2-MRG5. It contains five Gal4 binding sites immediately upstream of a synthetic HIV/AdML core promoter driving expression of a downstream luciferase cassette. Amplification primers were biotinylated 5'-GCATTCTAGTTGTGGTTTGTCCAA and 5'-ATACGACGATTCTGTGATTTG. Templates were purified on 1% (w/v) agarose gels, recovered using a gel extraction kit (Qiagen) and coupled to paramagnetic streptavidin beads (Promega, Madison, WI, USA) as follows: beads were washed twice in B&W buffer (5 mM Tris-HCl pH 7.5, 1 mM EDTA, 1 M NaCl, 0.003% Igepal CA-630). Subsequently, beads were resuspended in B&W buffer, and 15 ng biotinylated template (in 1× TE pH 8.0 containing 1 M NaCl) was added for each microgram of magnetic beads. After shaking for 45 minutes at room temperature, beads were washed once in B&W buffer containing 0.5 mg/ml BSA (fraction V; Sigma-Aldrich). For blocking, beads were resuspended at a concentration of 1 μg/μl in buffer A (60 mM KCl, 20 mM Hepes-KOH pH 8.2, 5 mM MgCl_2_, 10 mM dithiothreitol (DTT; Sigma-Aldrich), 0.025% Igepal CA-630, 0.2 mM phenylmethanesulfonyl fluoride (PMSF; Sigma-Aldrich)) containing 5 mg/ml BSA and 5 mg/ml polyvinylpyrrolidone (Sigma-Aldrich) and incubated for 15 minutes at room temperature. Afterwards, beads were washed three times with buffer A. PIC assembly was conducted in a total volume of 200 μl with 1,050 ng pGL2-MRG5 promoter template coupled to 70 μg beads, 2 μg poly(dG:dC) competitor DNA, 100 to 200 μg Jurkat nuclear extract and, if indicated, 200 ng Gal4-VP16 (the carboxy-terminal 147 amino acids of the *Saccharomyces cerevisiae *Gal4p DNA-binding domain linked to the Herpes simplex virus VP16 activation domain comprising residues 411 to 490). PIC assembly buffer was composed of 20 mM Hepes-KOH pH 8.2, 5 mM MgCl_2_, 10 mM DTT, 0.025% Igepal CA-630, 0.5 mg/ml BSA (Roche), 10% (w/v) glycerol, 0.1 mg/ml PEG 8000 and 0.2 mM PMSF. After 45 minutes incubation at 30°C the template-bound complexes were concentrated with a magnet and washed three times with 200 μl buffer A. PICs were either eluted with Laemmli buffer and analyzed by immunoblot or probed in an *in vitro *transcripton reaction (see below). Immunoblots were scanned and signal intensities quantified using the ImageJ program [41]. To test for the activity of template-associated PICs, *in vitro *transcription was performed. PICs were formed as above, washed and resuspended in transcription buffer (20 mM Hepes-KOH pH 8.2, 60 mM KCl, 5 mM MgCl_2_, 10 mM DTT, 0.025% Igepal CA-630 0.5 mg/ml BSA, 10% (w/v) glycerol, 0.1 mg/ml PEG 8000, 4 units RNAsin (Promega), 0.2 mM PMSF). Transcription was initiated by addition of the NTP mix supplemented with 1 μl alpha-^32^P UTP (3,000 Ci/mmol). Final NTP concentrations were 100 μM ATP, CTP and GTP each, and 5 μM UTP. Transcription reactions were incubated at 30°C for 30 minutes and stopped by addition of 400 μl transcription stop buffer (7 M urea, 10 mM Tris-HCl pH 7.8, 10 mM EDTA pH 8.0, 300 mM sodium acetate, 0.5% SDS, 100 mM lithium chloride, 0.4 mg/ml yeast tRNA). Reactions were extracted with phenol/chloroform, RNA precipitated with isopropanol and analyzed by autoradiography.

### Data accession

The raw and processed ChIP-chip data have been deposited at the Gene Expression Omnibus (GEO) public repository [42] and are accessible as [GEO:GSE19562]. Scaled log2 ChIP/input ratios of NimbleGen probeset signals from the TFIIB and NC2 ChIP-chip experiments are also available as Additional file 2.

## Abbreviations

AdML: Adenovirus major late; bp: base pair; BREd: downstream TFIIB recognition element; BREu: upstream TFIIB recognition element; BSA: bovine serum albumin; CAGE: cap analysis gene expression; ChIP: chromatin immunoprecipitation; ChIP-chip: ChIP with detection by microarrays; DPE: downstream promoter element; DTT: dithiothreitol; GAPDH: glyceraldehyde 3-phosphate dehydrogenase; GTF: general transcription factor; INR: initiator; NC2: negative cofactor 2 (DR1/DRAP1); PBS: phosphate-buffered saline; PEG: polyethylene glycol; PIC: preinitiation complex; PMSF: phenylmethanesulfonyl fluoride; qPCR: quantitative PCR; RNAPII: RNA polymerase II; TATA: TATA box; TBP: TATA box-binding protein; TFIIB: general transcription factor IIB (GTF2B); TSS: transcription start site.

## Authors' contributions

TKA and MM conceptualized the study; TKA and SB performed the experiments; TKA, KG and MM conducted the analyses; TKA and MM wrote the manuscript.

## Supplementary Material

Additional file 1 **Aligned core promoter regions of TFIIB target genes**. Nucleotide frequency plots of TSS-aligned core promoter regions from high-, low- and no-TFIIB genes. Click here for file

Additional file 2 **Data set of TFIIB and NC2 ChIP-chip signals**. Log2-scaled ChIP/input ratios of probeset signals from TFIIB and NC2 ChIP-chip experiments using two biological samples (Px5, chromatin from LCL721 B cells of passage 5; Px7, chromatin from LCL721 B cells of passage 7) on a NimbleGen human (HG17) promoter array. The data set includes the median, mean and standard deviation of 24,134 probesets, each composed of 15 individual 50-oligomer probes. Click here for file

## References

[B1] Lifton RP Goldberg ML Karp RW Hogness DS The organization of the histone genes in *Drosophila melanogaster *: functional and evolutionary implications. Cold Spring Harb Symp Quant Biol 1978 42 1047 1051 98262 10.1101/sqb.1978.042.01.105

[B2] Smale ST Baltimore D The "initiator" as a transcription control element. Cell 1989 57 103 113 10.1016/0092-8674(89)90176-1 2467742

[B3] Burke TW Kadonaga JT *Drosophila *TFIID binds to a conserved downstream basal promoter element that is present in many TATA-box-deficient promoters. Genes Dev 1996 10 711 724 10.1101/gad.10.6.711 8598298

[B4] Lewis BA Kim TK Orkin SH A downstream element in the human beta-globin promoter: evidence of extended sequence-specific transcription factor IID contacts. Proc Natl Acad Sci USA 2000 97 7172 7177 10.1073/pnas.120181197 10840054 PMC16518

[B5] Deng W Roberts SG A core promoter element downstream of the TATA box that is recognized by TFIIB. Genes Dev 2005 19 2418 2423 10.1101/gad.342405 16230532 PMC1257396

[B6] Lagrange T Kapanidis AN Tang H Reinberg D Ebright RH New core promoter element in RNA polymerase II-dependent transcription: sequence-specific DNA binding by transcription factor IIB. Genes Dev 1998 12 34 44 10.1101/gad.12.1.34 9420329 PMC316406

[B7] Lim CY Santoso B Boulay T Dong E Ohler U Kadonaga JT The MTE, a new core promoter element for transcription by RNA polymerase II. Genes Dev 2004 18 1606 1617 10.1101/gad.1193404 15231738 PMC443522

[B8] Juven-Gershon T Hsu JY Theisen JW Kadonaga JT The RNA polymerase II core promoter - the gateway to transcription. Curr Opin Cell Biol 2008 20 253 259 10.1016/j.ceb.2008.03.003 18436437 PMC2586601

[B9] Thomas MC Chiang CM The general transcription machinery and general cofactors. Crit Rev Biochem Mol Biol 2006 41 105 178 10.1080/10409230600648736 16858867

[B10] Saxonov S Berg P Brutlag DL A genome-wide analysis of CpG dinucleotides in the human genome distinguishes two distinct classes of promoters. Proc Natl Acad Sci USA 2006 103 1412 1417 10.1073/pnas.0510310103 16432200 PMC1345710

[B11] Sawadogo M Roeder RG Factors involved in specific transcription by human RNA polymerase II: analysis by a rapid and quantitative *in vitro *assay. Proc Natl Acad Sci USA 1985 82 4394 4398 10.1073/pnas.82.13.4394 3925456 PMC390420

[B12] Tsai FT Sigler PB Structural basis of preinitiation complex assembly on human pol II promoters. EMBO J 2000 19 25 36 10.1093/emboj/19.1.25 10619841 PMC1171774

[B13] Evans R Fairley JA Roberts SG Activator-mediated disruption of sequence-specific DNA contacts by the general transcription factor TFIIB. Genes Dev 2001 15 2945 2949 10.1101/gad.206901 11711430 PMC312826

[B14] Gershenzon NI Ioshikhes IP Synergy of human Pol II core promoter elements revealed by statistical sequence analysis. Bioinformatics 2005 21 1295 1300 10.1093/bioinformatics/bti172 15572469

[B15] Frith MC Valen E Krogh A Hayashizaki Y Carninci P Sandelin A A code for transcription initiation in mammalian genomes. Genome Res 2008 18 1 12 10.1101/gr.6831208 18032727 PMC2134772

[B16] Huisinga KL Pugh BF A TATA binding protein regulatory network that governs transcription complex assembly. Genome Biol 2007 8 R46 10.1186/gb-2007-8-4-r46 17407552 PMC1896006

[B17] Zanton SJ Pugh BF Changes in genomewide occupancy of core transcriptional regulators during heat stress. Proc Natl Acad Sci USA 2004 101 16843 16848 10.1073/pnas.0404988101 15548603 PMC534727

[B18] Venters BJ Pugh BF A canonical promoter organization of the transcription machinery and its regulators in the *Saccharomyces *genome. Genome Res 2009 19 360 371 10.1101/gr.084970.108 19124666 PMC2661807

[B19] Andrau JC Pasch L van de Lijnzaad P Bijma T Koerkamp MG Peppel J van de Werner M Holstege FC Genome-wide location of the coactivator mediator: Binding without activation and transient Cdk8 interaction on DNA. Mol Cell 2006 22 179 192 10.1016/j.molcel.2006.03.023 16630888

[B20] Zhu X Wiren M Sinha I Rasmussen NN Linder T Holmberg S Ekwall K Gustafsson CM Genome-wide occupancy profile of mediator and the Srb8-11 module reveals interactions with coding regions. Mol Cell 2006 22 169 178 10.1016/j.molcel.2006.03.032 16630887

[B21] van Werven FJ van Bakel H van Teeffelen HA Altelaar AF Koerkamp MG Heck AJ Holstege FC Timmers HT Cooperative action of NC2 and Mot1p to regulate TATA-binding protein function across the genome. Genes Dev 2008 22 2359 2369 10.1101/gad.1682308 18703679 PMC2532931

[B22] Albert TK Grote K Boeing S Stelzer G Schepers A Meisterernst M Global distribution of negative cofactor 2 subunit-alpha on human promoters. Proc Natl Acad Sci USA 2007 104 10000 10005 10.1073/pnas.0703490104 17548813 PMC1891239

[B23] Birney E Stamatoyannopoulos JA Dutta A Guigo R Gingeras TR Margulies EH Weng Z Snyder M Dermitzakis ET Thurman RE Kuehn MS Taylor CM Neph S Koch CM Asthana S Malhotra A Adzhubei I Greenbaum JA Andrews RM Flicek P Boyle PJ Cao H Carter NP Clelland GK Davis S Day N Dhami P Dillon SC Dorschner MO Fiegler H Identification and analysis of functional elements in 1% of the human genome by the ENCODE pilot project. Nature 2007 447 799 816 10.1038/nature05874 17571346 PMC2212820

[B24] Denissov S van Driel M Voit R Hekkelman M Hulsen T Hernandez N Grummt I Wehrens R Stunnenberg H Identification of novel functional TBP-binding sites and general factor repertoires. EMBO J 2007 26 944 954 10.1038/sj.emboj.7601550 17268553 PMC1852848

[B25] Kim TH Barrera LO Zheng M Qu C Singer MA Richmond TA Wu Y Green RD Ren B A high-resolution map of active promoters in the human genome. Nature 2005 436 876 880 10.1038/nature03877 15988478 PMC1895599

[B26] Zheng M Barrera LO Ren B Wu YN ChIP-chip: data, model, and analysis. Biometrics 2007 63 787 796 10.1111/j.1541-0420.2007.00768.x 17825010

[B27] Singh BN Hampsey M A transcription-independent role for TFIIB in gene looping. Mol Cell 2007 27 806 816 10.1016/j.molcel.2007.07.013 17803944

[B28] Crooks GE Hon G Chandonia JM Brenner SE WebLogo: a sequence logo generator. Genome Res 2004 14 1188 1190 10.1101/gr.849004 15173120 PMC419797

[B29] Bailey TL Elkan C Fitting a mixture model by expectation maximization to discover motifs in biopolymers. Proc Int Conf Intell Syst Mol Biol 1994 2 28 36 7584402

[B30] Gilfillan S Stelzer G Piaia E Hofmann MG Meisterernst M Efficient binding of NC2. TATA-binding protein to DNA in the absence of TATA. J Biol Chem 2005 280 6222 6230 10.1074/jbc.M406343200 15574413

[B31] Kawaji H Kasukawa T Fukuda S Katayama S Kai C Kawai J Carninci P Hayashizaki Y CAGE Basic/Analysis Databases: the CAGE resource for comprehensive promoter analysis. Nucleic Acids Res 2006 34 D632 636 10.1093/nar/gkj034 16381948 PMC1347397

[B32] Yudkovsky N Ranish JA Hahn S A transcription reinitiation intermediate that is stabilized by activator. Nature 2000 408 225 229 10.1038/35041603 11089979

[B33] Goppelt A Stelzer G Lottspeich F Meisterernst M A mechanism for repression of class II gene transcription through specific binding of NC2 to TBP-promoter complexes via heterodimeric histone fold domains. EMBO J 1996 15 3105 3116 8670811 PMC450253

[B34] Inostroza JA Mermelstein FH Ha I Lane WS Reinberg D Dr1, a TATA-binding protein-associated phosphoprotein and inhibitor of class II gene transcription. Cell 1992 70 477 489 10.1016/0092-8674(92)90172-9 1339312

[B35] Schluesche P Stelzer G Piaia E Lamb DC Meisterernst M NC2 mobilizes TBP on core promoter TATA boxes. Nat Struct Mol Biol 2007 14 1196 1201 10.1038/nsmb1328 17994103

[B36] Xie J Collart M Lemaire M Stelzer G Meisterernst M A single point mutation in TFIIA suppresses NC2 requirement *in vivo *. EMBO J 2000 19 672 682 10.1093/emboj/19.4.672 10675336 PMC305605

[B37] Roche NimbleGen http://www.nimblegen.com

[B38] Multivariate and Propensity Score Matching Software for Causal Inference http://sekhon.berkeley.edu/matching/

[B39] The R Project for Statistical Computing http://www.r-project.org

[B40] Boeing S Rigault C Heidemann M Eick D Meisterernst M RNAPII CTD SER-7 phosphorylation is established in a mediator-dependent fashion. J Biol Chem 2009 19901026 10.1074/jbc.M109.046565 PMC2804165

[B41] ImageJ http://rsbweb.nih.gov/ij/

[B42] Gene Expression Omnibus http://www.ncbi.nlm.nih.gov/geo/

[B43] Carninci P Sandelin A Lenhard B Katayama S Shimokawa K Ponjavic J Semple CA Taylor MS Engstrom PG Frith MC Forrest AR Alkema WB Tan SL Plessy C Kodzius R Ravasi T Kasukawa T Fukuda S Kanamori-Katayama M Kitazume Y Kawaji H Kai C Nakamura M Konno H Nakano K Mottagui-Tabar S Arner P Chesi A Gustincich S Persichetti F Genome-wide analysis of mammalian promoter architecture and evolution. Nat Genet 2006 38 626 635 10.1038/ng1789 16645617

[B44] Cage Basic Viewer http://fantom31p.gsc.riken.jp/cage/hg17/

